# Practical guide to implementing patient-reported outcome measures in gender-affirming care: evaluating acceptability, appropriateness and feasibility

**DOI:** 10.1136/bmjoq-2023-002677

**Published:** 2024-05-01

**Authors:** Rakhshan Kamran, Liam Jackman, Anna Laws, Melissa Stepney, Conrad Harrison, Abhilash Jain, Jeremy Rodrigues

**Affiliations:** 1 Nuffield Department of Orthopaedics, Rheumatology and Musculoskeletal Sciences, University of Oxford, Oxford, UK; 2 Temerty Faculty of Medicine, University of Toronto, Toronto, Ontario, Canada; 3 Northern Region Gender Dysphoria Service, Cumbria Northumberland Tyne and Wear NHS Foundation Trust, Newcastle Upon Tyne, UK; 4 Department of Psychiatry, University of Oxford, Oxford, UK; 5 Department of Plastic Surgery, Buckinghamshire Healthcare NHS Trust, Amersham, UK; 6 Warwick Clinical Trials Unit, University of Warwick, Coventry, UK

**Keywords:** Implementation science, Health services research, Health Equity, Healthcare quality improvement

## Abstract

**Objective:**

Assess acceptability, appropriateness and feasibility of the Practical Guide to Implementing patient-reported outcome measures (PROMs) in Gender-Affirming Care (PG-PROM-GAC) from a sample of patients and healthcare professionals.

**Design:**

Cross-sectional study conducted August–October 2023.

**Setting:**

Participants were recruited from a National Health Service (NHS) gender clinic.

**Participants:**

Patient participants seeking care and healthcare professionals working at an NHS gender clinic were eligible for participation. The PG-PROM-GAC was sent to participants via email for review.

**Outcome measures:**

Three validated tools to measure acceptability, appropriateness and feasibility were administered: the acceptability of intervention measure (AIM), intervention appropriateness measure (IAM) and feasibility of intervention measure (FIM). The percentage of participants indicating agreement or disagreement with items on the AIM, IAM and FIM was calculated.

**Results:**

A total of 132 transgender and gender diverse (TGD) patients (mean age, SD: 33, 14) and 13 gender-affirming healthcare professionals (mean age, SD: 43, 11) completed the AIM, IAM and FIM, representing a range of gender identities. The cumulative percentage of patients indicating agree or strongly agree on the AIM, IAM and FIM for the patient-relevant strategies in the PG-PROM-GAC was over 50% for each item. The cumulative percentage of patients indicating disagree or strongly disagree on the AIM, IAM and FIM for the PG-PROM-GAC was less than 20% for each item. The cumulative percentage of healthcare professionals indicating agree or strongly agree on the AIM, IAM and FIM for the healthcare professional-relevant strategies in the PG-PROM-GAC was over 38% for each item. The cumulative percentage of healthcare professionals indicating disagree or strongly disagree on the AIM, IAM and FIM for the PG-PROM-GAC was less than 15% for each item.

**Conclusions:**

Gender-affirming healthcare professionals and TGD patients find the PG-PROM-GAC acceptable, appropriate and feasible. The PG-PROM-GAC is ready-to-use for clinicians, policy-makers and researchers committed to service improvement for gender-affirming care.

**WHAT IS ALREADY KNOWN ON THIS TOPIC**

- Evidence-based implementation of patient-reported outcome measures (PROMs) for gender-affirming care is a priority, with a recent systematic review and qualitative study identifying that an evidence-based guide for PROM implementation in gender-affirming care is needed to help ensure uptake of PROMs for gender-affirming care.

**WHAT THIS STUDY ADDS**

- This study provides evidence of the acceptability, appropriateness and feasibility of the Practical Guide to Implementing PROMs in Gender-Affirming Care (PG-PROM-GAC) using validated measures and includes the perspectives of transgender and gender diverse (TGD) patients (n=132) and gender-affirming healthcare professionals (n=13).

**HOW THIS STUDY MIGHT AFFECT RESEARCH, PRACTICE OR POLICY**

- This study demonstrates that the PG-PROM-GAC is a ready-to-use tool for clinicians, researchers and policy-makers committed to improving gender-affirming care services through PROM implementation. The PG-PROM-GAC contains evidence-based strategies, which can be used to help guide PROM implementation, which has been developed and informed with feedback from TGD patients and ender-affirming healthcare professionals.

## Introduction

Patient-reported outcome measures (PROMs) are self-report questionnaires that measure how patients feel and function.^1^ When implemented well, PROMs can enhance communication between patients and providers, improve patient satisfaction, and patient outcomes.^2–4^ Yet, without effective implementation, the benefits of PROMs can be lost.^5^ This is a real issue—in some clinical settings, the rate of PROM use is fewer than 1% of what is possible, and this has been attributed to unaddressed implementation concerns.^6^

Gender-affirming care is one field where effective PROM implementation is a priority, to ensure measurement of patient outcomes and perspectives, in line with international clinical guidance.^7^ Gender-affirming care comprises a broad clinical area consisting of psychosocial, hormonal and surgical care aiming to ameliorate gender dysphoria.^7^ PROM implementation for gender-affirming care has the potential to drive patient-centred care, guide shared decision-making, challenge bias where appropriate and inform service development and improvement.^8^ However, PROM implementation for gender-affirming care worldwide is limited due to identified implementation barriers.^8 9^ Globally, evidence-based PROM implementation is urgently needed for gender-affirming care settings committed to improving patient outcomes and addressing continued issues with pathologising gender identity.^10–13^

The Practical Guide to Implementing PROMs in Gender-Affirming Care (PG-PROM-GAC) comprises evidence-based, patient-relevant and healthcare professional-relevant strategies developed to aid PROM implementation for gender-affirming care.^14^ The PG-PROM-GAC has been developed through a systematic review of 286 studies representing over 85 000 transgender and gender diverse (TGD) patients, a qualitative study of 14 TGD patients and 10 gender-affirming healthcare professionals, and iterative refinement with 7 TGD patients and 1 gender-affirming healthcare professional.^14^ The PG-PROM-GAC can be used to guide and maximise implementation of one of the 200+ identified PROMs available for specific needs in gender-affirming care settings.^14 15^ Before the PG-PROM-GAC can be used by clinicians and researchers, it is essential to evaluate its acceptability, appropriateness and feasibility.^16^

The aim of this study was to measure the acceptability, appropriateness and feasibility of the PG-PROM-GAC from a sample of TGD patients and healthcare professionals using the acceptability of intervention measure, intervention appropriateness measure (IAM) and feasibility of intervention measure (FIM).^17^

## Methods

### Patient and public involvement

Seven patients and members of the public representing individuals from the TGD community contributed to this research, including confirming the importance and relevance of the study question, and checking the applicability and relevance of findings. Patients and members of the public were recruited through local and national transgender charity and community organisations (ie, Gender Identity Research & Education Society, Oxford University LGBT+Advisory Groups).

### Reporting

Revised Standards for Quality Improvement Reporting Excellence 2.0 was followed for reporting this study.^18^

### Context

The healthcare context of this study is gender clinics in the UK National Health Service (NHS). Gender clinics can offer psychosocial, hormonal and surgical treatments, though this is dependent on a large range of factors such as degree of funding and investment in essential gender services.^12^

### Resource

The resource for this study is the PG-PROM-GAC,^14^ which comprises two tables with evidence-based strategies that might improve PROM uptake. One table focuses on patient-relevant strategies for PROM implementation and the other focuses on healthcare professional strategies for PROM implementation (online supplemental appendix 1).

10.1136/bmjoq-2023-002677.supp1 Supplementary data

### Study of the resource

Established theories, models and frameworks in implementation science were followed to study the PG-PROM-GAC. The aim of the PG-PROM-GAC is to improve PROM uptake as part of routine gender-affirming care. Therefore, normalisation process theory (NPT) was used to guide this study as NPT aims to understand how an innovation becomes routine.^19^ NPT was used to guide interpretation of results, and form conclusions and recommendations from this study. The knowledge to action (KTA) model was followed as it aims to guide translation of research into practice.^20^ The key step of the KTA model followed in this study is ‘evaluate outcomes’, which follows from previous steps of the KTA Model followed to develop the PG-PROM-GAC. The Consolidated Framework for Implementation Research was used to develop the PG-PROM-GAC.^21^ In line with guidance from implementation science, key implementation outcomes (acceptability, appropriateness and feasibility) were assessed in this study to study the PG-PROM-GAC.^16^

### Participants

Participants were recruited from an NHS gender clinic (The Northern Region Gender Dysphoria Service) in the UK. Patient participants seeking care and healthcare professionals working at an NHS gender clinic were eligible for participation and invited to participate via email.

### Measures

The AIM, IAM and FIM are each four-item measures, which can be administered to patients and healthcare professionals to determine the extent they believe a resource is acceptable, appropriate and feasible. The AIM, IAM and FIM have demonstrated acceptable psychometric properties in a series of three studies.^17^ Cut-off scores are not yet available for the AIM, IAM and FIM, however, higher scores (ie, indicating agree or strongly agree) indicate greater acceptability, appropriateness and feasibility.^17^ Items on the AIM, IAM and FIM were developed to be at the US fifth-grade reading level.

The AIM, IAM and FIM were administered to a sample of TGD patients (n=1859) and gender-affirming healthcare professionals (n=32) electronically on Microsoft Forms. Participants were invited to review the PG-PROM-GAC and complete the AIM, IAM and FIM. Participants were sent a reminder email 1 week after the initial study invitation was sent. The duration of data collection was 2 weeks.

### Analysis

Descriptive frequencies were used to analyse demographic information of the study sample (ie, age, gender identity). The proportion of individuals responding to each response option (completely disagree, disagree, neither agree nor disagree, agree, completely agree) for the items of the AIM, IAM and FIM were calculated. Analyses were conducted by using SPSS (V.28.0.1) software.

## Results

### Demographic information for participants

A total of 132 TGD patients (mean age, SD: 33, 14) and 13 gender-affirming healthcare professionals (mean age, SD: 43, 11) completed the AIM, IAM and FIM, with no missing data. The TGD patient sample ranged in age from 18 to 71. The gender-affirming healthcare professional sample ranged in age from 23 to 57. Most patients and healthcare professionals were white (124, 94% and 11, 85%, respectively) with ethnic/racialised minorities represented at 4% for the patient sample and 15% for the healthcare professional sample. Most participants in the patient and healthcare professional sample were British (112, 85% and 11, 85%, respectively). Most patient participants were female (57, 43%) versus male (39, 30%) gender and had sex assigned at birth of female (61, 46%) versus male (70, 53%). Most healthcare professionals were female (11, 85%) with a female sex assigned at birth (9, 69%) versus male (4, 31%). Table 1 demonstrates the demographic information for this study sample.

**Table 1:** Demographic information of study sample*

TGD patient characteristics	
Demographic information	Frequency (%)
Age (mean, SD) (n=131)	33 (14)
Gender	
Demigirl	1 (1)
Female	57 (43)
Genderqueer	1 (1)
Genderfluid	2 (2)
Male	39 (30)
Multigender	1 (1)
Nonbinary	6 (5)
Queer	1 (1)
Trans female	8 (6)
Trans femme	1 (1)
Trans male	8 (6)
Trans masculine	3 (2)
Transgender	1 (1)
NR	3 (2)
Sex assigned at birth	
Female	61 (46)
Male	70 (53)
NR	1 (1)
Race	
Asian	3 (2)
Mixed—white and black	1 (1)
Mixed—European	1 (1)
Mixed—white and Asian	1 (1)
White	124 (94)
NR	2 (2)
Ethnicity	
American	1 (1)
British	112 (85)
British and Chinese	2 (2)
British and Irish	3 (2)
British and Japanese	1 (1)
British and European	1 (1)
Celtic	1 (1)
Irish	3 (2)
Mixed European	1 (1)
Norwegian	1 (1)
Pakistani	1 (1)
Welsh and British	1 (1)
NR	4 (3)
Healthcare professional characteristics	
Demographic information	Frequency (%)
Age (mean, SD) (n=13)	43 (11)
Gender	
Female	11 (85)
Genderqueer femme	1 (8)
Male	1 (8)
Sex assigned at birth	
Female	9 (69)
Male	4 (31)
Race	
Asian	1 (8)
Mixed—white and Asian	1 (8)
White	11 (85)
Ethnicity	
British	11 (85)
Chinese	1 (8)
European	1 (8)

*NR indicates where data were not available as participants did not share this information.

TGD, transgender and gender diverse.

### Acceptability of the PG-PROM-GAC

The cumulative percentage of patients indicating agree and strongly agree to each item of the AIM for the patient-relevant strategies (approving the patient-relevant strategies, finding the patient-relevant strategies appealing, liking the patient-relevant strategies and welcoming the patient-relevant strategies) are 52%, 52%, 52% and 56%, respectively. The cumulative percentage of patients indicating agree and strongly agree to each item of the AIM for the healthcare professional-relevant strategies (approving the healthcare professional-relevant strategies, finding the healthcare professional-relevant strategies appealing, liking the healthcare professional-relevant strategies and welcoming the healthcare professional-relevant strategies) are 41%, 42%, 39% and 46%, respectively. The cumulative percentage of patients indicating disagree and strongly disagree with each item of the AIM for the PG-PROM-GAC was under 22% for each item.

**Figure 1:**
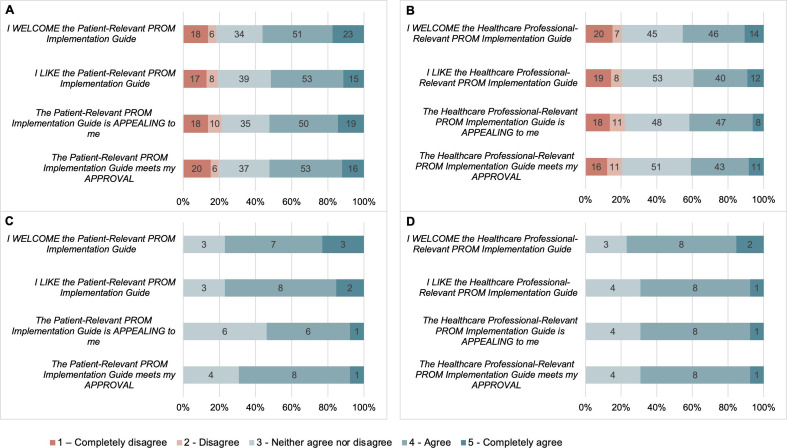
Acceptability of PG-PROM-GAC. (A) Patient Acceptability of Patient- Relevant PROM Implementation Guide. (B) Patient Acceptability of Healthcare Professional-Relevant PROM Implementation Guide. (C) Healthcare Professional Acceptability of Patient-Relevant PROM Implementation Guide. (D) Healthcare Professional Acceptability of Healthcare Professional-Relevant PROM Implementation Guide. PG-PROM-GAC, Practical Guide to Implementing PROMs in Gender-Affirming Care; PROMs, patient-reported outcome measures.

The cumulative percentage of healthcare professionals indicating agree and strongly agree to each item of the AIM for the patient-relevant strategies (approving the patient-relevant strategies, finding the patient-relevant strategies appealing, liking the patient-relevant strategies, and welcoming the patient-relevant strategies) are 69%, 54%, 54% and 77%, respectively. The cumulative percentage of healthcare professionals indicating agree and strongly agree to each item of the AIM for the healthcare professional-relevant strategies (approving the healthcare professional-relevant strategies, finding the healthcare professional-relevant strategies appealing, liking the healthcare professional-relevant strategies and welcoming the healthcare professional-relevant strategies) are 69%, 69%, 69% and 77%, respectively. No healthcare professional participants indicated disagreement on the AIM for the patient-relevant or healthcare professional-relevant PROM implementation strategies. Figure 1 graphically displays the breakdown of responses to the AIM from both patients and healthcare professionals.

### Appropriateness of the PG-PROM-GAC

The cumulative percentage of patients indicating agree and strongly agree to each item of the IAM for the patient-relevant strategies (the patient-relevant strategies seem fitting, the patient-relevant strategies seem suitable, the patient-relevant strategies seem applicable, the patient-relevant strategies seem like a good match) are 55%, 57%, 54% and 51%, respectively. The cumulative percentage of patients indicating agree and strongly agree to each item of the IAM for the healthcare professional-relevant strategies (the healthcare professional-relevant strategies seem fitting, the healthcare professional-relevant strategies seem suitable, the healthcare professional-relevant strategies seem applicable, the healthcare professional-relevant strategies seem like a good match) are 44%, 43%, 44% and 43%, respectively. The cumulative percentage of patients indicating disagree and strongly disagree with each item of the AIM for the PG-PROM-GAC was under 22% for each item.

**Figure 2:**
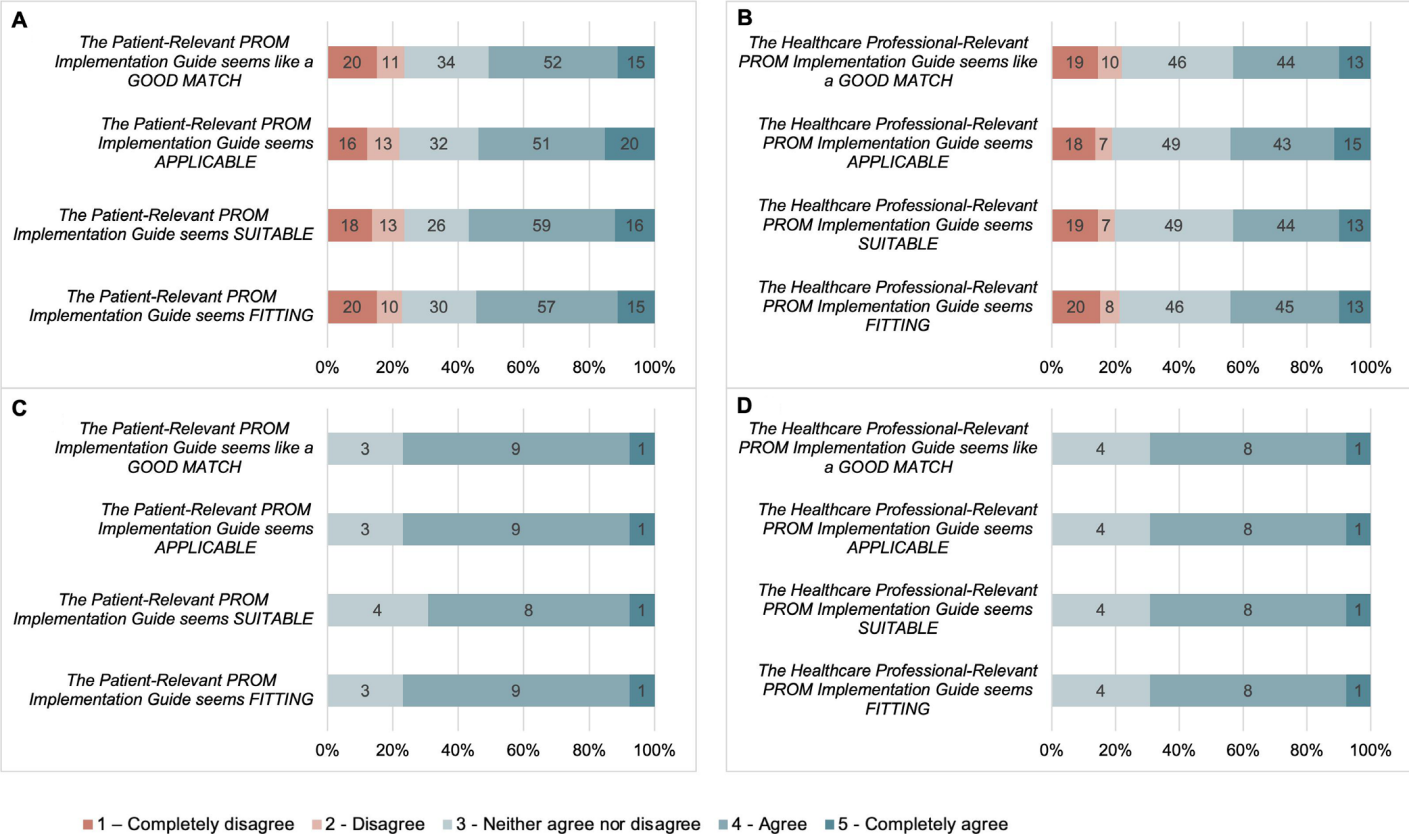
Appropriateness of the PG-PROM-GAC. (A) Patient Appropriateness of Patient-Relevant PROM Implementation Guide. (B) Patient Appropriateness of Healthcare Professional-Relevant PROM Implementation Guide. (C) Healthcare Professional Appropriateness of Patient-Relevant PROM Implementation Guide. (D) Healthcare Professional Appropriateness of Healthcare Professional-Relevant PROM Implementation Guide. PG-PROM-GAC, Practical Guide to Implementing PROMs in Gender-Affirming Care; PROMs, patient-reported outcome measures.

The cumulative percentage of healthcare professionals indicating agree and strongly agree to each item of the IAM for the patient-relevant strategies (the patient-relevant strategies seem fitting, the patient-relevant strategies seem suitable, the patient-relevant strategies seem applicable, the patient-relevant strategies seem like a good match) are 77%, 69%, 77% and 77%, respectively. The cumulative percentage of healthcare professionals indicating agree and strongly agree to each item of the IAM for the healthcare professional-relevant strategies (the healthcare professional-relevant strategies seem fitting, the healthcare professional-relevant strategies seem suitable, the healthcare professional-relevant strategies seem applicable, the healthcare professional-relevant strategies seem like a good match) are 69% for each item. No healthcare professional participants indicated disagreement on the IAM for the patient-relevant or healthcare professional-relevant PROM implementation strategies. Figure 2 graphically displays the breakdown of responses to the IAM from both patients and healthcare professionals.

### Feasibility of the PG-PROM-GAC

The cumulative percentage of patients indicating agree and strongly agree to each item of the FIM for the patient-relevant strategies (the patient-relevant strategies seem implementable, the patient-relevant strategies seem possible, the patient-relevant strategies seem doable, the patient-relevant strategies seem easy to use) are 56%, 59%, 57% and 50%, respectively. The cumulative percentage of patients indicating agree and strongly agree to each item of the FIM for the healthcare professional-relevant strategies (the healthcare professional-relevant strategies seem implementable, the healthcare professional-relevant strategies seem possible, the healthcare professional-relevant strategies seem doable, the healthcare professional-relevant strategies seem easy to use) are 44%, 46%, 43% and 41%, respectively. The cumulative percentage of patients indicating disagree and strongly disagree with each item of the FIM for the PG-PROM-GAC was under 20% for each item.

**Figure 3:**
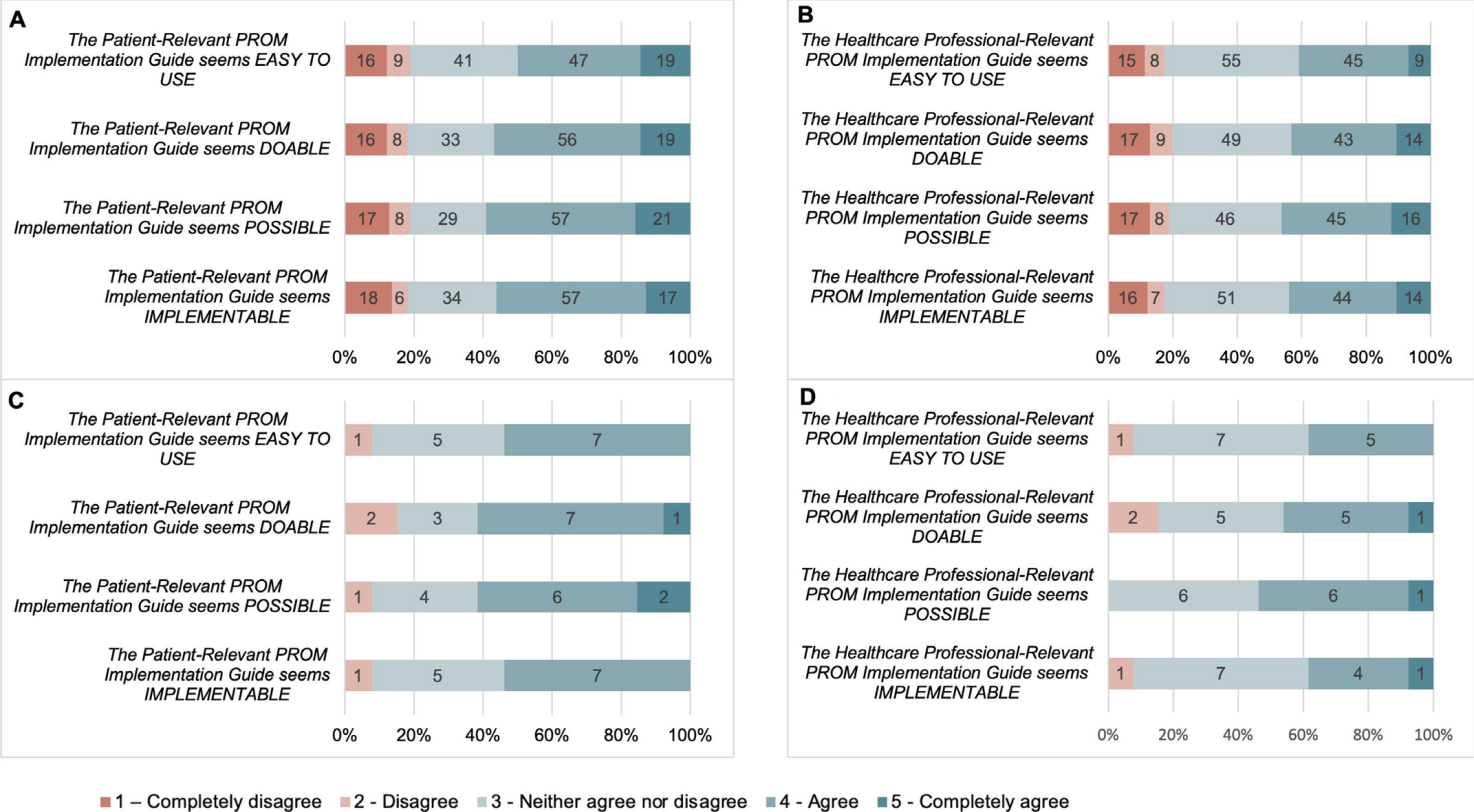
Feasibility of the PG-PROM-GAC. (A) Patient Appropriateness of Patient-Relevant PROM Implementation Guide. (B) Patient Appropriateness of Healthcare Professional-Relevant PROM Implementation Guide. (C) Healthcare Professional Appropriateness of Patient-Relevant PROM Implementation Guide. (D) Healthcare Professional Appropriateness of Healthcare Professional-Relevant PROM Implementation Guide. PG-PROM-GAC, Practical Guide to Implementing PROMs in Gender-Affirming Care; PROMs, patient-reported outcome measures.

The cumulative percentage of healthcare professionals indicating agree and strongly agree to each item of the FIM for the patient-relevant strategies (the patient-relevant strategies seem implementable, the patient-relevant strategies seem possible, the patient-relevant strategies seem doable, the patient-relevant strategies seem easy to use) are 54%, 62%, 62% and 54%, respectively. The cumulative percentage of healthcare professionals indicating agree and strongly agree to each item of the FIM for the healthcare professional-relevant strategies (the healthcare professional-relevant strategies seem implementable, the healthcare professional-relevant strategies seem possible, the healthcare professional-relevant strategies seem doable, the healthcare professional-relevant strategies seem easy to use) are 38%, 54%, 46% and 38%, respectively. The cumulative percentage of healthcare professionals indicating disagree and strongly disagree with each item of the FIM for the PG-PROM-GAC was under 15% for each item. Figure 3 graphically displays the breakdown of responses to the FIM from both patients and healthcare professionals.

## Discussion

This study establishes the acceptability, appropriateness and feasibility of the PG-PROM-GAC based on a sample of 132 TGD patients and 13 gender-affirming healthcare professionals. The PG-PROM-GAC can be used by clinicians, policy-makers and researchers to implement PROMs for their gender-affirming care setting to improve PROM uptake and lead evidence-based PROM implementation initiatives.

In general, there were some discrepancies between patient and healthcare professional results. When patient participants were asked about the healthcare professional-relevant PROM implementation strategies, there was a high rate of ambivalence (individuals indicating they neither agree nor disagree), compared with when patients were responding to patient-relevant strategies. A potential reason for this could be patient participants feeling the healthcare professional-relevant strategies were not relevant for them to provide feedback on. Additionally, healthcare professionals were more positive about both patient and healthcare professional PROM guides, apart from feasibility, where there was a lower rate of agreement on the FIM items. A potential reason for this is the perceived burden of implementing certain strategies from the PG-PROM-GAC. In order to investigate in-depth the reasons why participants may have scored the way they did, our team is currently conducting a qualitative study.

Evidence-based PROM implementation strategies are a key component to realising the benefits of PROMs.^5 22 23^ In a study of PROM implementation for palliative care, it was identified that having a coordinator through the implementation process and an educational component prior to implementation was key to implementation success.^24^ Our PG-PROM-GAC identifies education as a key implementation strategy as well as identifying implementation champions who can help facilitate implementation, in line with the results from this study. In an article on PROM implementation for total knee arthroplasty, a key implementation strategy outlined was the electronic administration of PROMs.^25^ In our implementation guide, we also identified strategies such as multifactor authentication when using PROMs electronically to ensure data security, and linking PROM responses to electronic medical records to enhance their clinical utility. In a study of PROM implementation for breast cancer care, ‘challenges with completing PROMs’ were identified as a key barrier to successful PROM implementation.^26^ Our implementation guide addresses this identified barrier with a recommended strategy for healthcare professionals to consider burden of PROM scoring when selecting a PROM to use for their setting.

Past systematic reviews conducted by our team identified a total of 205 PROMs used for gender-affirming care, with 38 PROMs used for paediatric gender-affirming care.^8 15^ Development of new PROMs is time-intensive and resource-intensive and may contribute to research waste.^27^ It is reported that in order to use healthcare funding and resources more efficiently, existing PROMs identified for certain clinical settings should be validated to meet current standards rather than the creation of entirely new PROMs.^27 28^ The PG-PROM-GAC can be used to help clinicians and researchers implement a PROM they identify as most useful for their setting and can apply to versions of PROMs currently available and their future iterations as they undergo further validation.

Strengths of this study include the inclusion of a study sample diverse in gender identity, the use of validated implementation outcome measures, and that this study followed established theories, methods and frameworks in implementation science. Further, we conducted this research in partnership with patients and members of the public to ensure the relevance and importance of this study, and relevance and applicability of findings. Finally, the results from this study demonstrate that the PG-PROM-GAC is a ready-to-use tool for clinicians and researchers globally. In the UK, in particular, PROM implementation is urgently needed in gender identity clinics that require systemic reform to address continued issues of pathologising gender identity and poor patient experience.^10–13^ Gender clinics worldwide committed to improving patient experience could benefit from the guide, as it demonstrates acceptability, and appropriateness, and feasibility.

There are some limitations to consider. First, there was a lack of racial and ethnic diversity in this study sample. Future testing of the PG-PROM-GAC should seek the perspectives of racial and ethnic minority groups not represented enough in this study sample. Second, participants in this sample were from the UK (predominantly from the Northern region of England). Although the PG-PROM-GAC was developed from evidence which includes a worldwide systematic review, future studies should seek the perspectives of TGD patients and healthcare professionals from countries in addition to the UK. The scope of the present study was the UK and this is an opportunity for researchers in other contexts to adapt this work. Third, we were unable to compare the characteristics of participants in our study with the characteristics of the total target population. This was due to being unable to compare demographic information of participants who did not consent to participate in this study. It is possible that this could limit the generalisability of our results to the total target population. Future research should seek to address this limitation and identify potential demographic differences in gender clinic populations versus study populations. Fourth, this study measured implementation outcomes quantitatively and did not consider qualitative feedback from TGD patients and gender-affirming healthcare professionals. Therefore, qualitative measurement of thoughts, opinions and feedback for the PG-PROM-GAC was not captured in this study. To overcome this limitation, our team is conducting a qualitative study of TGD patients and healthcare professionals on their feedback for the PG-PROM-GAC.^29^ Finally, there is a need to actually implement, use, and evaluate the PG-PROM-GAC. The strength of our study from an implementation science perspective is that we have done an a priori analysis of acceptability, appropriateness and feasibility. When implementing, it is important to compare these results with experience in the real-world setting and then iteratively improve the PG-PROM-GAC to make sure that it indeed succeeds at doing what it was designed to do. Research from our team is underway to overcome this limitation.^30^

## Conclusion

This study demonstrates the acceptability, appropriateness and feasibility of the PG-PROM-GAC as a ready-to-use tool for clinicians, policy-makers and researchers aiming to implement PROMs for their gender-affirming care setting or leading their own outcome measurement and evaluation initiatives for gender-affirming care.

## Data Availability

Data are available on reasonable request. Reasonable requests for data can be made to the corresponding author and with a data transfer agreement, if applicable.
